# Effect of Mobile Phase Additives on the Resolution of Four Bioactive Compounds by RP-HPLC

**DOI:** 10.3390/ijms11052229

**Published:** 2010-05-25

**Authors:** Shengnan Li, Minglei Tian, Kyung Ho Row

**Affiliations:** Department of Chemical Engineering, Inha University, Incheon 402-751, Korea; E-Mails: lishengnank@hotmail.com (S.L.); feitiandezhu@hotmail.com (M.T.)

**Keywords:** mobile phase additive, bioactive compounds, ionic liquids, *Herba Artemisiae Scopariae*

## Abstract

The use of mobile phase additives enhances the separation and resolution of the bioactive compounds on the C_18_ column. Chlorogenic acid, caffeic acid, rutin, and scoparone from *Herba Artemisiae Scopariae* were investigated as the target compounds. Acetic acid, triethylamine, inorganic salts, and several ionic liquids were added as mobile phase additives into methanol/water (40:60, v/v). The result revealed that a mobile phase with 0.01 mol/L of ionic liquid [BMIM][BF_4_] enabled the optimum separation of the four target compounds.

## 1. Introduction

**Figure 1.**
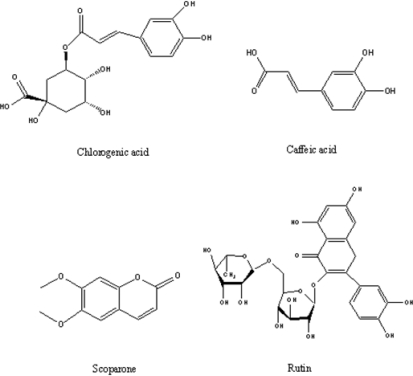
The chemical structures of the four target compounds.

*Herba Artemisiae Scopariae* (HAS) is one of the oldest medicinal herbs in traditional Chinese medicine [1]. HAS has been shown to have various pharmacological effects, such as liver protection, blood pressure lowering, antipyretic, anti-inflammatory, antibacterial, anti-microbial and antitumor activity [2–5]. It has been used to treat acute icteric infectious hepatitis, hyperlipemia, and oral ulcers. Chlorogenic acid, caffeic acid, scoparone and rutin (the structures are shown in Figure 1) are the four major active components of the herb. It should be noted that the amount of chlorogenic acid (0.15% of total sample weight) is often used as the criteria of quality control for HAS [6,7].

In order to separate these bioactive compounds by HPLC, a C_18_ column was used. Methanol, was used as the most common organic solvent in the mobile phase [8,9]. Unfortunately, such simple binary systems (methanol/water eluents) do not satisfy the needs of analysts in the vast majority of cases [10]. The majority of separations are impossible without various mobile phase additives, such as acids, salts, and organic compounds. Variation of the nature and the concentration of organic modifiers affects the interaction energy of the analyte in the bulk mobile phase. In recent years, interest in new modifiers has grown rapidly [11,12].

Ionic liquids (ILs) are a type of salt that are liquid at low temperatures (<293 K). In recent years, ILs have been successfully applied in various areas of analytical chemistry, catalysis, and others. ILs have a variety of desirable properties, such as negligible vapor pressure, good thermal stability, tunable viscosity, and miscibility with water and organic solvents [13]. In addition, it is anticipated that ILs can be applied to sample preparation procedures [14–21].

**Figure 2.**
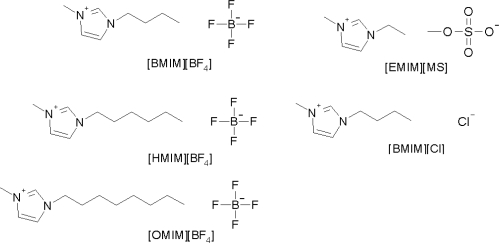
The chemical structures of the ionic liquids used as mobile phase additives.

There are some previously published reports about the separation of chlorogenic acid, caffeic acid, scoparone and rutin [22–25]. However, there is no research about the effect of the mobile phase additives for the simultaneous separation of these four compounds. In this study, methanol/water (40:60, v/v) was fixed as the mobile phase. Several additives, such as acetic acid, triethylamine, and ionic liquids (1-butyl-3-methylimidazolium tetrafluoroborate ([BMIM][BF_4_]), 1-hexyl-3-methylimidazolium tetrafluoroborate ([HMIM][BF_4_]), 1-methyl-3-octylimidazolium tetrafluoroborate ([OMIM][BF_4_]), 1-ethyl-3-methylimidazolium methlsulfate ([EMIM][MS]), and 1-butyl-3-methylimidazolium chloride ([BMIM][Cl]), were evaluated as mobile phase additives (Figure 2) for the separation of the mixture of the four compounds from HAS by RP-HPLC. The effects of the type and concentration of additives on the chromatographic performance are discussed.

## 2. Results and Discussion

### 2.1. Retention Factor Estimation

In this work the retention factor was calculated according to equation (1).

(1) $$k=(t_{R}-t_{0})/t_{0}$$

where *t_0_* is the hold-up time, *t_R_* is the retention time, and *k* is retention factor. The resolution (*R*) is calculated using the eq. (2).

(2) $$R=2(t_{R2}-t_{R1})/(w_{1}+w_{2})$$

where *t_R1_* and *t_R2_* are the retention times of the first and second peak, respectively, and *w_1_* and *w_2_* are the peak widths at the baselines of the first and second peaks, respectively.

(3) $$\text{Related}\;\text{erro}=(1-k_{a}/k_{ave}\text{)}\times 100\%$$

where *k_a_* is the retention factor for each target compound and *k_ave_* is the average retention factor for each target compound with several injections.

### 2.2. Effect of Acid and Basic Additives on the Resolution

**Figure 3.**
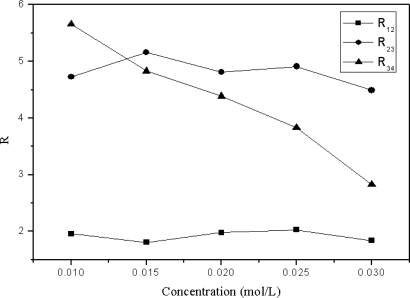
Effect of the concentration of acetic acid on the resolution of the four compounds. (Error bars smaller than the symbols are not shown). 1: chlorogenic acid, 2: caffeic acid, 3: scoparone, 4: rutin; R_12_ = resolution of chlorogenic acid and caffeic acid; R_23_ = resolution of caffeic acid and scoparone; R_34_ = resolution of scoparone and rutin.

**Figure 4.**
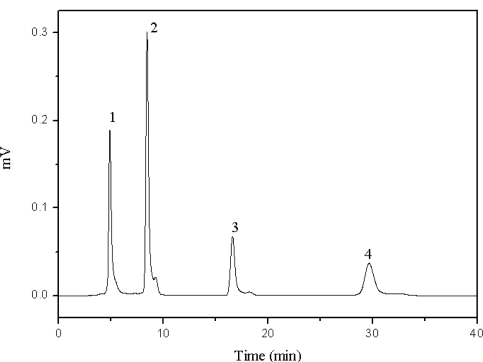
Chromatogram of the four target compounds with 0.01 mol/L of acetic acid as the mobile phase additive. 1: chlorogenic acid, 2: caffeic acid, 3: scoparone, 4: rutin.

The acidity or basicity of the mobile phase can have a significant influence on separation. Acetic acid and triethylamine, as two traditional mobile phase additives in reverse phase HPLC, were employed to establish acidic or alkaline conditions, respectively. Chlorogenic acid and caffeic acid are organic acids which contain hydroxyl and carboxyl groups. Thus, acetic acid had more efficiency in increasing the resolution of these two compounds than triethylamine (Table 1). Furthermore, the interactions between target compounds and the stationary phase were reduced with increasing concentrations of acetic acid in the mobile phase. In this case, the resolution of the four target compounds was decreased (Figure 3). Moreover it should be noted that high concentrations of acid in the mobile phase may be harmful for the HPLC system. Therefore, a slightly acidic mobile phase was found to be the most suitable for the separation of the four compounds (Figure 4). The presence of a shoulder on the chromatographic peaks can be explained by the difference between the sample and the mobile phase solvents.

### 2.3. Effect of the Concentration of Inorganic Salt

**Figure 5.**
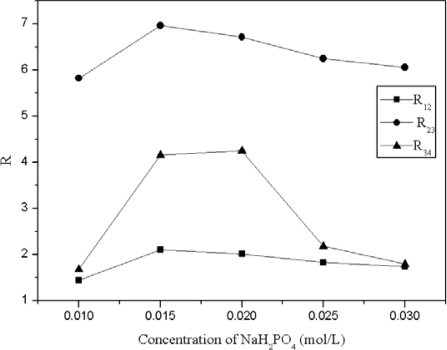
Effect of concentration of NaH_2_PO_4_ on the resolution of the four compounds. (Error bars smaller than the symbols are not shown). 1: chlorogenic acid, 2: caffeic acid, 3: scoparone, 4: rutin; R_12_= resolution of chlorogenic acid and caffeic acid; R_23_= resolution of caffeic acid and scoparone; R_34_= resolution of scoparone and rutin.

**Figure 6.**
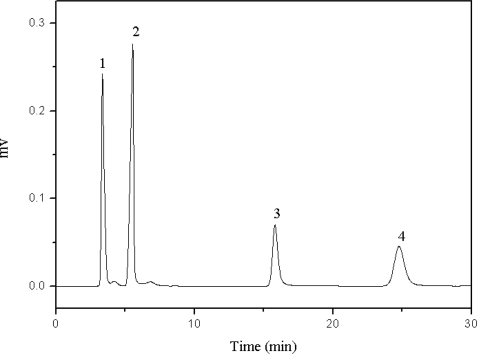
Chromatogram of the four target compounds with 0.02 mol/L of NaH_2_PO_4_ as the mobile phase additive. 1: chlorogenic acid, 2: caffeic acid, 3: scoparone, 4: rutin.

Next, the effect of inorganic salts was evaluated. When Na_2_HPO_4_ was added, the mobile phase showed basicity and the target compounds could not be separated (Table 1). The retention factors of the four target compounds increased with increasing concentrations of NaH_2_PO_4_ until 0.02 mol/L, after, which the retention factors decreased. This result reveals that at a low concentration of NaH_2_PO_4_ in mobile phase, ion exchange interaction between cations and anions play a major role in aqueous solution. With this interaction between target compounds and the surface of the stationary phase, the retention time was increased. When more NaH_2_PO_4_ was added, the acidity of the mobile phase was increased because of the high concentration of hydrogen ion. At this point, the interaction between target compounds and the stationary phase was decreased and the retention times of target compounds were reduced. The resolution of the four compounds via NaH_2_PO_4_ as the additive is shown in Figure 5. The figure shows that 0.02 mol/L was the optimum concentration (Figure 6).

### 2.4. Effect of Ionic Liquid on the Retention and Resolution of Target Compounds

As mobile phase additives, organic solvents have animal toxicity; additionally, inorganic buffers are potentially harmful for the HPLC system and the reverse-phase column. In this case, ionic liquid, which has organic cations and anions, was used.

#### 2.4.1. Different Cations of Ionic Liquid

**Figure 7.**
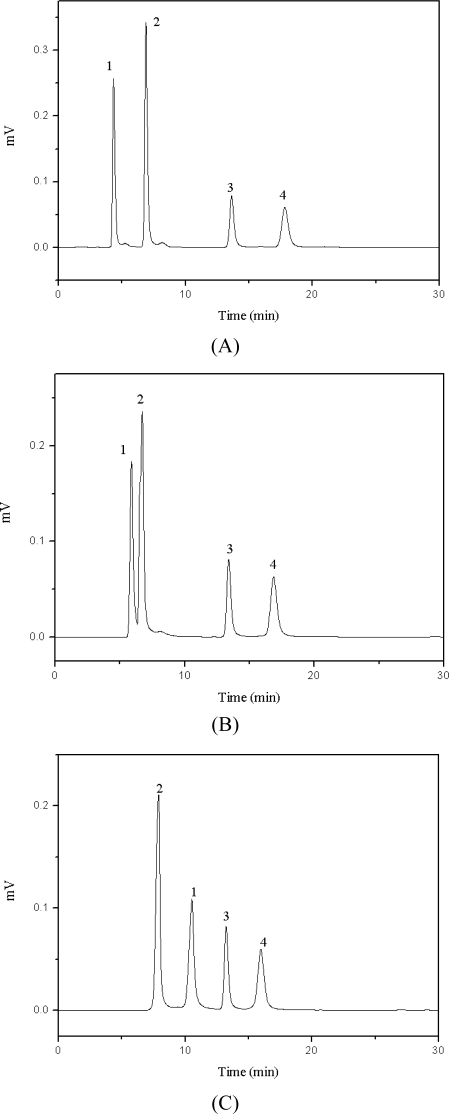
Chromatograms of the four target compounds with 0.01 mol/L of ionic liquid as the mobile phase additives. (A) [BMIM][BF_4_], (B) [HMIM][BF_4_], (C) [OMIM][BF_4_]. 1: chlorogenic acid, 2: caffeic acid, 3: scoparone, 4: rutin.

The cations of the three ionic liquids have different carbon chains; each chain has a particular hydrophobic characteristic. Figure 7 shows three chromatograms of the four target compounds under mobile phase with three different ionic liquids. The interaction between C_18_ and ionic liquid increased with increasing hydrophobicity of the ionic liquid. Thus the retention time was increased in accordance with the electrostatic interaction of ionic liquid and chlorogenic acid. Therefore, [BMIM][BF_4_] and [OMIM][BF_4_] were suitable to be used as the additives.

Furthermore, the resolution of the four compounds went from 2.2 to 2.5, and from 1.2 to 2.9, respectively, when the concentrations of [BMIM][BF_4_] and [OMIM][BF_4_] increased from 0.01 mol/L to 0.03 mol/L. By comparing the total retention time and resolution of mobile phases that contain acetic acid, NaH_2_PO_4_, and [BMIM][BF_4_] as additives, it can be determined that the four compounds can be obtained with optimized resolution via the usage of a mobile phase with [BMIM][BF_4_] as the additive at the low concentration.

#### 2.4.2. Effect of Anion [MS] of Ionic Liquid

**Figure 8.**
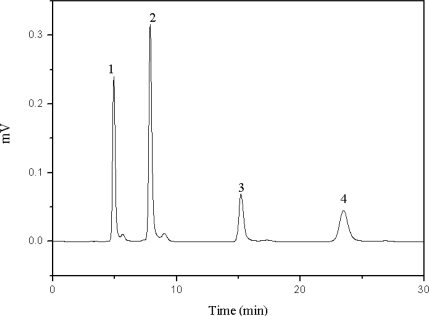
Chromatogram of the four target compounds with 0.01 mol/L of [EMIM][MS] as the mobile phase additive. 1: chlorogenic acid, 2: caffeic acid, 3: scoparone, 4: rutin.

**Figure 9.**
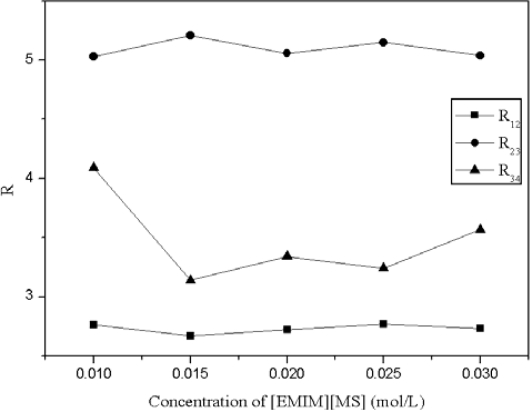
Effect of concentration of [EMIM][MS] on the resolution of the four compounds. (Error bars smaller than the symbols are not shown). 1: chlorogenic acid, 2: caffeic acid, 3: scoparone, 4: rutin; R_12_= resolution of chlorogenic acid and caffeic acid, R_23_= resolution of caffeic acid and scoparone, R_34_= resolution of scoparone and rutin.

Zheng *et al.* [26] reported that the aqueous mobile phase showed acidity when 0.03 mol/L of [EMIM][MS] was used as the additive. In Figure 8, the target compounds can be obtained via base-line separation by using a mobile phase with 0.01 mol/L of [EMIM][MS]. Figure 9 shows that the resolution slightly decreased with an increasing concentration of [EMIM][MS]. The total retention time of [EMIM][MS] was longer than that of other ionic liquids, but the total retention time was less than that of acetic acid and NaH_2_PO_4_. Additionally, the resolution was higher with acetic acid and NaH_2_PO_4_. The results proved that the use of ionic liquids as additives are better than the use of alternative additives and that the optimized condition for these ionic liquids in mobile phase was with 0.01 mol/L [BMIM][BF_4_].

#### 2.4.3. Effect of Anion of Ionic Liquid

The effect of different anions of ionic liquids was established by comparing [BMIM][BF_4_] with [BMIM][Cl]. Because of the hydrogen bond accepting ability of the chlorine, the Cl^−^ anion disrupted the hydrogen bonding between target compounds and the stationary phase [27]. Thus, the two organic acid compounds cannot be separated by using [BMIM][Cl] as the additive (Table 2).

## 3. Experimental Section

### 3.1. Materials and Reagents

Standards of chlorogenic acid, caffeic acid, scoparone, and rutin were purchased from the National Institute for the Control of Pharmaceutical and Biological Products (Beijing, China). Methanol, triethylamine, Na_2_HPO_4_, NaH_2_PO_4_, and acetic acid were obtained from the Duksan Pure Chemical Co., LTD (Ansan, Korea). Ionic liquids were offered by the Chem Tech Research Incorparation (Hwaseong, Korea). All the other reagents used in the experiment were HPLC or analytical grade. Double distilled water was filtered with a vacuum pump (Division of Millipore, Waters, U.S.A.) and filter (HA-0.45, Division of Millipore, Waters, U.S.A.) before use.

### 3.2. Preparation of Mobile Phases and Standard Solutions

Acetic acid, triethylamine, inorganic salt and ionic liquids were added into the methanol/water (40:60, v/v) solution directly. The molar concentrations were adjusted from 0.01 mol/L to 0.03 mol/L. All the mobile phase solvents were mixed completely for further experiments. Stock solutions of chlorogenic acid, caffeic acid, scoparone, and rutin were dissolved in methanol to make a final concentration of 25.0 μg/mL. It should be emphasized that the working solutions were re-prepared every 3 days so as to avoid potential errors arising from decomposition.

### 3.3. Apparatus and Chromatographic Conditions

Chromatography was performed with a Waters 600s multisolvent delivery system, a Waters 616 liquid chromatography, and a Waters 2487 variable wavelength, dual-channel UV detector (Waters Associates, Milford, MA, USA). A six-port Rheodyne injector (20-μL sample loop) was also used. Data processing was performed with Millennium 3.2 software resident in an HP Vectra 500PC. Compounds were separated on a 150 mm × 4.6 mm, 5-μm particle, OptimaPak C_18_ column (RStech, Daejeon, Korea). The flow rate was set at 0.5 mL/min. The wavelength was fixed at 325 nm. An injection volume of 10 μL was applied throughout the experiments. All procedures were carried out at ambient temperature.

Individual compounds and mixtures of standard compounds were injected 5 times to confirm the retention times and scientific replications.

## 4. Conclusions

In this research, the effects of acetic acid, triethylamine, inorganic salts, and several ionic liquids as mobile phase additives were investigated by using four bioactive compounds. All the additives influenced two organic acids in the target compounds. Excellent separation condition was obtained with methanol/water (40:60, v/v) and 0.01 mol/L [BMIM][BF_4_] as the additive. In any case, ionic liquids show promising performance as additives in HPLC.

## Figures and Tables

**Table 1.** Effect of concentrations of different mobile phase additives on retention factors. CGA: chlorogenic acid; CA: caffeic acid; SC: scoparone.

**Concentration (mol/L)**	**Compound**	***k*(error, %)**			
		**Acetic acid**	**Triethylamine**	**NaH_2_PO_4_**	**Na_2_HPO_4_**
0.01	CGA	1.61 (0.62)	0.12 (0.05)	0.66 (0.60)	0.51 (0.06)
	CA	3.51 (0.52)		1.51 (0.57)	
	SC	7.88 (0.63)	5.83 (0.42)	6.32 (0.6)	7.49 (0.73)
	Rutin	14.86 (1.82)	0.12 (0.05)	7.99 (0.98)	0.51 (0.06)
0.015	CGA	1.57 (0.60)	0.12 (0.05)	0.80 (0.67)	0.53 (0.06)
	CA	3.34 (0.81)		1.96 (0.49)	
	SC	7.53 (0.65)	5.53 (0.43)	7.45 (0.86)	6.88 (0.68)
	Rutin	13.24 (1.74)	0.12 (0.05)	12.24 (1.36)	0.53 (0.06)
0.02	CGA	1.55 (0.62)	0.12 (0.05)	0.84 (0.67)	0.56 (0.06)
	CA	3.25 (0.55)		2.09 (0.58)	
	SC	7.35 (0.62)	4.85 (0.42)	7.59 (0.90)	6.63 (0.70)
	Rutin	12.39 (1.25)	0.12 (0.05)	12.63 (0.99)	0.56 (0.06)
0.025	CGA	1.51 (0.61)	0.12 (0.05)	0.81 (0.60)	0.58 (0.063)
	CA	3.12 (0.56)		1.86 (0.54)	
	SC	7.11 (0.66)	4.59 (0.40)	6.81 (0.58)	6.59 (0.66)
	Rutin	11.44 (1.12)	0.12 (0.05)	9.64 (0.87)	0.58 (0.06)
0.03	CGA	1.49 (0.43)	0.12 (0.05)	0.82 (0.60)	0.60 (0.07)
	CA	2.99 (0.47)		1.81 (0.56)	
	SC	6.85 (0.58)	4.30 (0.40)	6.58 (0.74)	7.93 (0.82)
	Rutin	10.20 (1.10)	0.12 (0.05)	8.49 (0.86)	1.03 (0.83)

**Table 2.** Effect of the concentration of the different ionic liquids on retention factors.

**Concentration (mol/L)**	**Compound**	***k*(error, %)**				
		**[BMIM][BF_4_]**	**[HMIM][BF_4_]**	**[OMIM][BF_4_]**	**[EMIM][MS]**	**[BMIM][Cl]**
0.01	CGA	1.20 (0.75)	2.12 (0.80)	4.63 (1.00)	1.63 (0.80)	0.94 (1.50)
	CA	2.67 (0.73)	2.54 (0.69)	3.22 (0.83)	3.22 (0.83)	
	SC	6.51 (0.72)	6.07 (0.66)	6.08 (0.82)	7.16 (0.81)	6.13 (0.91)
	Rutin	9.41 (1.20)	7.74 (1.18)	7.54 (1.18)	11.66 (1.09)	7.03 (0.96)
0.015	CGA	1.29 (0.75)	2.18 (0.80)	4.47 (0.95)	1.57 (0.80)	0.98 (1.50)
	CA	2.86 (0.73)	2.95 (0.69)	3.28 (0.83)	3.01 (0.83)	
	SC	6.76 (0.74)	6.71 (0.69)	6.38 (0.82)	6.95 (0.79)	6.60 (0.77)
	Rutin	10.22 (1.23)	9.72 (1.16)	8.00 (1.21)	10.28 (1.10)	8.06 (0.99)
0.02	CGA	1.30 (0.75)	2.28 (0.79)	4.20 (0.86)	1.55 (0.80)	0.93 (1.40)
	CA	2.91 (0.72)	2.90 (0.72)	3.29 (0.88)	2.98 (0.70)	
	SC	6.74 (0.70)	6.73 (0.70)	6.50 (0.67)	6.72 (0.72)	4.65 (0.58)
	Rutin	10.17 (1.18)	9.63 (1.17)	8.15 (1.09)	10.13 (1.05)	6.12 (0.87)
0.025	CGA	1.21 (0.72)	2.29 (0.79)	5.09 (0.92)	1.52 (0.80)	0.93 (1.40)
	CA	2.43 (0.66)	2.84 (0.71)	3.50 (0.89)	2.94 (0.70)	
	SC	5.88 (0.58)	6.61 (0.68)	6.70 (0.68)	6.63 (0.67)	5.47 (0.92)
	Rutin	7.21 (0.99)	9.11 (0.98)	9.50 (1.18)	9.90 (1.13)	6.43 (0.83)
0.03	CGA	1.33 (0.75)	2.32 (0.79)	4.43 (0.86)	1.56 (0.81)	0.93 (1.30)
	CA	2.68 (0.66)	2.94 (0.71)	3.32 (0.88)	3.04 (0.72)	
	SC	6.30 (0.67)	6.64 (0.68)	6.48 (0.66)	6.80 (0.65)	3.93 (0.47)
	Rutin	8.54 (1.08)	8.63 (0.96)	7.36 (0.91)	10.57 (1.05)	6.07 (0.78)
